# Surgery of Simple and Complex Anal Fistulae in Adults: A Review of the Literature for Optimal Surgical Outcomes

**DOI:** 10.7759/cureus.35888

**Published:** 2023-03-08

**Authors:** Anestis Charalampopoulos, Dimitrios Papakonstantinou, George Bagias, Konstantinos Nastos, Markos Perdikaris, Savvas Papagrigoriadis

**Affiliations:** 1 Third Department of Surgery, "Attikon" University General Hospital/National and Kapodistrian University of Athens, School of Medicine, Athens, GRC; 2 Rectal and Pelvic Surgery, Metropolitan General Hospital Athens, Athens, GRC

**Keywords:** surgery, endorectal advancement flap, laser fistulotomy, video-assisted anal fistula treatment, fistulotomy, fistulectomy, complex anal fistula, simple fistula, fistula-in-ano, anal fistula

## Abstract

Anal fistulas are common anorectal conditions, and surgery is the primary treatment option. In the last 20 years of literature, there exist a large number of surgical procedures, especially for the treatment of complex anal fistulas, as they present more recurrences and continence problems than simple anal fistulas. To date, there are no guidelines for choosing the best technique. We conducted a recent literature review, mainly the last 20 years, based on the PubMed and Google Scholar medical databases, with the goal of identifying the surgical procedures with the highest success rates, lowest recurrence rates, and best safety profiles. Clinical trials, retrospective studies, review articles, comparative studies, recent systematic reviews, and meta-analyses for various surgical techniques, as well as the latest guidelines of the American Society of Colon and Rectal Surgeons, the Association of Coloproctology of Great Britain and Ireland, and the German S3 guidelines on simple and complex fistulas were reviewed.

According to the literature, there is no recommendation for the optimal surgical technique. The etiology, complexity, and many other factors affect the outcome. In simple intersphincteric anal fistulas, fistulotomy is the procedure of choice. In simple low transsphincteric fistulas, the patient’s selection is crucial in order to perform a safe fistulotomy or another sphincter-saving technique. The healing rate in simple anal fistulas is higher than 95% with low recurrence and without significant postoperative complications. In complex anal fistulas, only sphincter-saving techniques should be used; the optimal outcomes are obtained by the ligation of the intersphincteric fistulous tract (LIFT) and rectal advancement flaps. Those techniques assure high healing rates of 60-90%. The novel technique of the transanal opening of the intersphincteric space (TROPIS) is under evaluation. The novel sphincter-saving techniques of fistula laser closure (FiLac) and video-assisted anal fistula treatment (VAAFT) are safe, with reported healing rates ranging from 65% to 90%. Surgeons should be familiar with all sphincter-saving techniques in order to face the variability of the fistulas-in-ano. Currently, there is no universally superior technique that can treat all fistulas.

## Introduction and background

An anal fistula (AF) is an abnormal communication between the anorectal canal and the peri-anal skin. It is part of the natural history of the perianal abscess. The main characteristics of a fistula are the following: (a) the presence and location of the internal opening (IO) at the anorectal canal, (b) the presence of an external opening at the anal/perianal skin, and (c) the presence of a fistulous tract of various length and route affecting the anorectal sphincter system: internal, external, and levator ani (puborectalis) muscles. Secondary tracts and abscess cavities connected with the fistulous tract are not excluded.

Even though AF is a common condition in anorectal surgery, it is a rare disease [1] with a prevalence of fewer than 5 cases/10,000 per 10,000 people. The most common etiologies are cryptoglandular AF and the second most common anorectal Crohn’s disease (CD). Other etiologies, such as iatrogenic trauma, infections, and malignancy, present a lower incidence and prevalence.

The pathogenesis of AF still remains unclear; in cryptoglandular AF [2], histological, microbiological, molecular, and host factors are related to the development and persistence of AF, while in anorectal CD, the trans-mural inflammatory process contributes to the formation of anorectal abscess and AF. Cryptoglandular fistulas should be distinguished from secondary etiologies due to differences in surgical outcomes, which mainly depend on the underlying disease. AF affects more middle-aged patients with a male-to-female ratio of 2/1. To date, the only acceptable cutting-sphincter surgical procedure in use is fistulotomy in simple AF. In complex AF, many sphincter-saving procedures are in use for the treatment of AF, and the guidelines leave several options.

Studies that assess the treatment of cryptoglandular AF present heterogeneity [3], without uniform outcomes, and the comparisons between different procedures are quite difficult, but some surgical procedures seem to be more superior to other techniques.

The aim of this review study is to identify the range of surgical procedures in use, the factors that affect the surgeons’ choice, and also to collect data for the surgical procedures in use regarding the surgical outcomes and the safety of the procedure, regarding the postoperative continence or other anal local complications. We identify the most important, currently in-use surgical procedures for AF treatment from a large spectrum of surgical procedures available. We provide data for successful individual therapy in patients with AF and a strategy in surgery for optimal outcomes, based on the recent literature.

## Review

Complexity systems of classification

There are many systems of classification of AF in the literature; the most common and important ones in use are described here. Park’s system of classification [4], in place since 1976, belongs to the era before the widespread use of imaging. It is a clinical/surgical system of classification of cryptoglandular fistulas that defines AF into four main types: intershincteric, transsphincteric, suprasphincteric, and extrasphincteric fistulas; the submucosal AF (simple entities without anal sphincter participation in fistula formation) were not included in the original classification due to different etiologies; this classification has many variations and references to other more complex entities. The most common AF are intersphincteric and transsphincteric AF. The grade and types of fistulas in this classification system are described in Table 1.

**Table 1:** The Parks system classification for anal fistulas Supralevator fistula could be present in grade I, II, or III. Translevator fistula could be present in grade II (IIb) or IV (IVa).

Grade	Description of the fistula	Characteristics
I	Intersphincteric	Ia: low; I_b_: high extension in rectal wall without an additional high opening in the rectum; I_c_: high extension in rectal wall with an additional high opening in the rectum; I_d_: no external opening in the perianal skin in the type Ib or Ic; I_e_: high extension in pelvic cavity; I_f_: pelvic disease draining into the perianal skin through the intersphincteric space
II	Transsphincteric	IIa: all fistulas below the puborectalis muscle; II_b_: fistula with a branch going high in ischiorectal fossa (infralevator) or high through levator muscle (translevator) but not opening into the rectum
III	Suprasphincteric	Suprasphincteric fistula with or without a supralevator extension
IV	Extrasphincteric	IV_a_: transsphincteric fistula with a branch going high through levator muscle (translevator) but opening into the rectum (type IIb with an additional opening high in the rectum); IV_b_: extrasphincteric tract due to trauma; IV_c_: extrasphincteric tract due to anorectal disease like Crohn’s disease, ulcerative colitis, or carcinoma; IV_d_: pelvic disease draining into the perianal skin after piercing through the levator muscle

Another system of classification that belongs to the post-magnetic resonance imaging (MRI) era is the St. James’s University Hospital (SJUH) system of classification. It is a descriptive system, and most radiologists are familiar with the above system [5]. Based on the MRI evaluation of AF and the spectrum of imaging features, AF was classified into five grades of complexity with an allocation of predictive value to MRI for postoperative outcomes, according to secondary tracts and abscesses that increase the grade of the complexity of AF. Grades I and II are simple fistulas with favorable surgical outcomes, and grades III and IV are the complex transsphincteric AFs with more recurrences after surgery and an increased possibility for incontinence problems. Grade V is the most complex AF; those are the translevator or supralevator, which are surgical challenges but fortunately have a low incidence. The main characteristics of AF are shown in Table 2.

**Table 2:** The St. James’s University Hospital classification of anal fistulas by MRI Suprasphincteric fistulas was categorized along transsphincteric fistulas as grade IV.

Grade	Description of the fistula
I	Intersphincteric: linear
II	Intersphincteric: multiple tracts or associated abscess
III	Transsphincteric: linear
IV	Transsphincteric: multiple tracts or associated abscess
V	Supralevator or translevator/extrasphincteric

In a recent study based on a large number of patients who underwent surgery for cryptoglandular AF, a modified version of Park’s system classification [6] distinguishes AF into four stages of simple and complex AF, incorporating: (a) changes in the description of AF; those transsphincteric were divided into low and high transsphincteric AF, while suprasphincteric and extrasphincteric (internal opening of AF at the rectum) were grouped into one type, (b) four independent risk factors were significant predictors of surgical failure; secondary AF extensions, horseshoe AF, previous AF surgery, and anterior AF in women. The first three types of AF were subdivided according to the presence or absence of predictive factors for surgical failure. Thus, the failure rates in surgery were ranging from 2.3% in simpler AF up to 30.7% in more complex AF. The modified Parks classification system is shown in Table 3.

**Table 3:** The modified Parks classification system for crypto-glandular anal fistulas

Stage	Characteristics
I	Intersphincteric fistula not involving the external anal sphincter fibers
IA	Simple linear, non-branching intersphincteric tract
IB	Intersphincteric tract with at least one of the following: Horseshoe tract, secondary extensions and associated abscess cavities, anterior fistula in female patients, history of previous surgery for anal fistula
II	Low transsphincteric fistula involving less than 30% of external anal sphincter fibers
IIA	Simple linear, non-branching low transsphincteric tract
IIB	Low transsphincteric tract with at least one of the following: Horseshoe tract, secondary extensions and associated abscess cavities, anterior fistula in female patients, history of previous surgery for anal fistula
III	High transsphincteric fistula involving more than 30% of external anal sphincter fibers
IIIA	Simple linear non-branching high transsphincteric tract
IIIB	High transsphincteric tract with at least one of the following: Horseshoe tract, secondary extensions and associated abscess cavities, anterior fistula in female patients, history of previous surgery for anal fistula
IV	Unusual types of fistula: suprasshincteric and extrasphincteric

Another new system of classification for AF was proposed by Garg [7], based on a large number of patients. It seems to be more accurate than Park’s and SJUH's classification of AF [8] and classifies the severity of AF in five main grades with important implications for further surgical management; all patients are classified according to the preoperative MRI and the severity of AF [9]. The benefit is the correct classification of AF in simple AF, where fistulotomy is a safe operation, and in more complex AF, where fistulotomy is contraindicated and a sphincter-saving technique should be used. This system contains five grades of complexity, etiology, and risk factors and provides treatment guidelines. The original system of classification is shown in Table 4.

**Table 4:** The Garg P. original classification system for anal fistulas FPR: fistulotomy with primary reconstruction, EAS: external anal sphincter, AFP: anal fistula plug.

Grade	Treatment guidelines
I: I-A: low-linear intersphincteric; I-B: low-linear transsphincteric (less than 1/3 of EAS involvement).	Fistulotomy should be possible in >95% of these AF.
II: low intersphincteric and transsphincteric AF (less than 1/3 of EAS involvement); II-A: abscess; II-B׃ multiple tracts; II-C׃ horseshoe; II-D׃ supralevator: complete intersphincteric supralevator AF; II-E: supralevator: Low transsphincteric (<1/3 EAS involvement) with intersphincteric supralevator extension.	Fistulotomy should be possible >90% of these AF.
III: III-A: high linear transsphincteric fistula (>1/3 EAS involvement); III-B: fistula with associated Crohn’s disease, sphincter injury, post-radiation exposure or anterior fistulae in a female.	Fistulotomy should not be attempted. FPR or sphincter-saving procedures: LIFT, VAAFT, AFP, TROPIS, OTSC, or FiLac therapy should be done.
IV: complex high (>1/3 EAS involvement). Transsphincteric fistula with either: IV-A: abscess; IV-B: multiple tracts; IV-C: Horseshoe.	Fistulotomy should not be attempted. FPR or sphincter-saving procedures: LIFT, VAAFT, AFP, TROPIS, OTSC, or FiLac therapy should be done. Preferably refer these AF to a fistula expert.
V: V-A: transsphincteric (>1/3 EAS Involvement) with intersphincteric supralevator extension; V-B: suprasphincteric fistula; V-C: extrasphincteric fistula.	Fistulotomy should not be attempted. FPR or sphincter-saving procedures: LIFT, VAAFT, AFP, TROPIS, OTSC, or FiLac therapy should be done. Preferably refer these AF to a fistula expert.

The characteristics of simple and complex anal fistulas

From a practical standpoint, all systems of classification of AF for diagnosis and further surgical management describe two distinct conditions. The simple AF, where the fistulotomy is a safe operation with high healing rates and no postoperative continence problems if the patients are correctly selected and classified, and the complex AF, with significant participation of the anal sphincter muscle in the fistula formation, present more recurrences after surgery and continence problems; thus, in surgery, only sphincter-saving procedures should be used. Complex AF is the transsphincteric AF with the participation of the external sphincter of more than 30% in fistula formation (the most common complex AF), suprasphincteric AF, extraspincteric AF, horseshoe AF, recurrent AF, anterior AF in women, AF in relation to inflammatory bowel diseases, pelvic radiation, and malignancy.

Studying the complexity of AF

Simple AF probably does not require preoperative imaging studies if the preoperative diagnosis is accurate. All other AF, such as complex AF, anal CD, recurrent AF, immunosuppressive patients, and patients with occult anal abscess or AF, according to the recommendations of the American Society of Colon and Rectal Surgeons (ASCRS) [10], should be studied preoperatively by imaging studies.

The most useful investigation is an MRI with the fistula protocol. MRI is the optimal preoperative imaging study [11], more sensitive than clinical evaluation, and comparable with endoanal ultrasounds (EUS) in the distinction between simple and complex AF. A practical MRI radiologic report [12] provides surgeons with all necessary information for the location of the internal opening of the AF, the external opening location, the classification of AF, the presence of secondary tracts and abscesses, the evaluation of the supralevator space, the presence or not of a previous sphincter injury, and the activity of the fistulous tract; it may be active (with fluid or pus within the tract) or more fibrotic. This information has significant implications for further surgical management. The assessment of the ischio-anal and ischio-rectal fossa [13] by the presence of a fistulous tract reveals a complex AF of grade III or IV (transsphincteric or suprasphincteric); indeed, if the fistulous tract traverses the lavatory ani muscle, it shows a more complex AF of grade V (supralevator or translevator).

The endoanal three-dimensional ultrasound scan (3D EUS) or 2D has a supplementary role because of its potential to be used multiple times for follow-up with the economy of costs and time resources. Three-dimensional EUS and MRI [14] are accurate in simple AF; the results are comparable in complex AF; and MRI is superior to EUS in the detection of secondary tracts.

Examination under anesthesia (EUA) is the traditional gold standard of assessment for the proctologist, which is important for the choice of optimum surgical management. EUA in anal CD should be performed before the medical therapy [15], as the procedure gives the opportunity to drain an abscess or to place drainage setons for the improvement of the local anal inflammatory process. EUA is the gold standard procedure for the evaluation of anal CD [16], and EUA with MRI or EUS presents 100% diagnostic accuracy [17] for the evaluation of anal CD.

Outcomes of surgery and the current practice of anal surgeons

Many factors are related to surgical outcomes [18], such as the experience of the surgeon, the complexity of AF, the involvement of the anal sphincter muscles, the method of the surgical procedure, and many patient-related factors.

On the other hand, the position of the anal surgical community presents significant differences not only in the choice of surgical technique but also in the diagnostic procedures used for the evaluation of AF [19]. In a recent international survey for the surgical practice and management of AF with a 74-item questionnaire, there were broad technique variations in surgical practice, and it was difficult to reproduce and compare the outcomes between different centers. Here are some interesting results of the study. Some 80% of respondents consider fistulotomy the gold standard treatment for simple anal fistulas. The ligation of the intersphincteric fistulous tract (LIFT) procedure with technical variations is performed by 38% of surgeons. When an endorectal advancement flap is performed, full-thickness flaps are more commonly used than partial-thickness flaps. Novel techniques such as video-assisted anal fistula treatment (VAAFT), fistula laser closure (FiLac), and over-the-scope clips (OTSC) were used by less than 10% of the respondents. Only 1-4% of surgeons were confident enough to perform one of the novel sphincter-saving techniques in patients with anal CD.

Surgery of simple AF

Surgery is the only treatment option in AF, whereas in CD, where the anal canal is affected in 20-40% of patients, medical agents contribute to the remission of the disease [20]; combined surgical therapy by drainage setons with immunomodulators and anti-tumor necrosis factor (TNF) may contribute to AF closure.

To date, the only acceptable sphincter-cutting procedure in use is fistulotomy in simple AF. It is probably the most common operation in use, as simple AF accounts for 30-50% of all AF. The healing rates are more than 95%.

It is a safe and easy operation with continence preservation and low recurrence rates depending on the presence of secondary tracts, the identification of the IO, and the etiology (cryptoglandular or anal CD).

In simple low transsphincteric fistulas (or low intersphincteric, as they were called in the past; defined as grade II AF in MRI or low AF) containing less than 30% of the external anal sphincter, a fistulotomy may be complicated by symptoms of incontinence [21], and the risk of impairment continence is one to five patients; most patients present minor continence problems, but more severe problems are not excluded and are dependent from the amount of external sphincter divided during fistulotomy. It is unknown what amount of the external sphincter is divided that influences continence, but it seems that a division of the external anal sphincter of more than 25% [22] is correlated with a high Fecal Incontinence Severity Index score after fistulotomy. Continence problems may be transient or persistent. Thus, fistulotomy is not completely harmless, as one to five patients will present continence problems; this percentage probably is unacceptable in the era of numerous sphincter-saving procedures, and patients should be carefully selected for a safe fistulotomy.

The correct distinction between a simple intersphincteric fistula and a low transsphincteric fistula, with the evaluation of the amount of external sphincter participating in fistula formation, is crucial for the choice of the surgical technique; the choice is between fistulotomy and another sphincter-saving procedure.

Another key point in the successful treatment of a simple AF is the integrity of the anal sphincteric system, is the intersphincteric space alone affected, or is part of a more complex sphincter system involved? In the former case, fistulotomy is adequate, and in the latter case, a sphincter-saving procedure for complex AF should be performed.

To date, fistulotomy in simple AF has gained its position in the guidelines of the ASCRS, the German S3 guidelines [23], the Italian Society of Colorectal Surgery (SICCR) [24], and in the second Association for Coloproctology of Great Britain and Ireland (ACPGBI) position statement for the treatment of AF [25], with strong recommendations with level evidence 1B, 1B, 2B, and C, respectively. A careful selection of patients is crucial to performing a safe fistulotomy.

Surgery of complex AF

In complex AF, only sphincter-saving procedures should be used. The goal of surgery is first to remove or destroy the fistulous tract while preserving the integrity of the sphincters, and then to identify risk factors for the recurrence of AF.

The outcomes of surgery in complex AF present more recurrences and continence problems than in surgery of simple AF. Numerous factors are reported in the literature as risk factors for recurrence after surgery [26]. They are factors related to AF anatomy and other anal comorbidities (previous anal surgery and eventual sphincter damage, anal CD, post-radiotherapy AF), numerous preoperative and intraoperative factors, and factors related to postoperative complications and care.

In a recent meta-analysis [27], many factors of major or minor significance for recurrence are described; high-risk factors for recurrence are a high transsphincteric fistula, a missed IO at surgery, and horseshoe extensions. This evidence comes from high-quality observational studies. Other risk factors associated with the recurrence of AF are the presence of secondary tracts, prior anal surgery, and seton placement.

In a case-series study with 483 patients about the long-term results of surgical treatment of AF [28], the recurrence rate for complex AF with various surgical procedures was 18%, with a requirement of up to three reoperations before complete healing.

In the last 20 years, several surgical procedures have been used for complex AF, some of them popular and others less so. In the literature, the surgical outcomes, healing rates, recurrence rates, and incontinence rates vary between studies for any specific technique; heterogeneity in the etiology and grade of the complexity of the AF in patients studied, as well as the methodology of studies, explain the differences in surgical outcomes. The majority of the studies are retrospective, case-series, or non-randomized control studies with various inclusion criteria.

In a systematic review and meta-analysis of surgical interventions for high crypto-glandular AF [29], the best surgical technique could not be identified, and there was a need for more randomized control trials. Surgeons should be familiar with several procedures available for AF treatment in order to be able to choose the most suitable surgical technique after imaging and classification. At the moment, a universally optimal surgical procedure cannot be identified.

Most common operations for complex anal fistulas

Cutting Seton

The Hippocratic technique was more popular in the past, but it is now practiced less [30] due to reported high rates of incontinence. In the previous meta-analysis (since 2009), the incontinence rates varied from 12% to 30% in transsphincteric AF and 53% in suprasphincteric AF. The recent recommendation for the technique in selected patients is weak with evidence 2C, according to the practice guidelines of the ASCRS. There have been no studies in the last 10 years on the current use of the method in complex AF.

Drainage (Loose) Seton

This is useful and often necessary in the acute phase of anal/perianal sepsis. It helps to improve local clinical symptoms and downstage the complexity of the AF. Drainage setons may stay for weeks, months, or years according to the local evolution of sepsis. They are commonly used in fistulizing anorectal CD control in combination with medical agents. The failure of surgical or medical therapy with refractory anal fistulizing symptoms may require eventual radical surgical therapy with proctectomy and permanent colostomy. There are no new publications to provide recommendations for the method in practice guidelines, except in fistulizing anal CD for long-term control of the disease (strong recommendation, evidence 1B).

Plugs in Fistula

They are minimally invasive techniques; however, they have healing rates [31,32] in complex AF of 50% or less one year after surgery. The successful healing rates decrease with time. Plugs are not an adequate treatment for complex AF.

Glue Sealants

There is an increased failure of healing [33] with time after the performance of this procedure; the healing rates are 14%, 16 months after surgery. There have been no new publications on the method in the last 10 years. The method may be used only in exceptional cases, according to the German S3 guidelines for AF.

In both procedures, glue sealants and plugs remain in use only for selected patients and only in combination with other sphincter-saving procedures.

Rectal Advancement Flaps (RAF)

The procedure includes the curettage of the fistulous tract, the closure of the IO with sutures, and its obliteration by a rectal flap [34]. Partial-thickness RAF of any shape, whether rhomboid or elliptical, is the most common in use, and the shape does not influence the failure rates. The failure rates in the literature vary from 15 to 60%. The majority of patients postoperatively present with some degree of incontinence. The procedure is repeatable if the first mucosal RAF fails, and it may be used in recurrences after other sphincter-saving techniques, e.g., LIFT.

In a systematic review and meta-analysis [35] of the use of RAF and ligation of the intersphincteric fistula tract (LIFT) for cryptoglandular AF and anal CD, RAF had similar and comparable outcomes with LIFT regarding the healing rates, but incontinence was statistically significant in RAF more than in LIFT, with a lack of data for recurrences.

In another review and systemic meta-analysis [36] of RAF in complex cryptoglandular AF, the pooled rate of recurrence was 21%, with rates of 7.4%, 19%, and 30.1% for full-thickness, partial, and mucosal flaps, respectively. Indeed, all flaps caused some incontinence, which was increased by the flap's thickness. RAF and LIFT may be used to treat AF in anal CD.

In the most recent ASCRS guidelines, evidence 1B has a strong recommendation for treating RAF. 

LIFT

This procedure, with many variations, has gained popularity in the last 10 years as it is easily performed, safe, cheap, without significant postoperative incontinence, and has high healing rates of 60-90%. In a simplified variation, the main surgical steps [37] are the division and ligation with sutures of the fistulous tract at the level of the intersphincteric space. Failure of the procedure does not exclude a repeat operation. The procedure may be used in both complex and simple low transsphincteric AF.

In a review study and meta-analysis [38], with the majority of patients (92.36%) having transsphincteric AF and long-term follow-up, LIFT showed favorable short- and long-term outcomes, a low postoperative complications rate of 1.88%, an overall mean healing rate of 81.37%, an overall healing period of 8.15 weeks, and AF recurrence in 7.58% of patients in the studied reports.

In a recent meta-analysis [39] with a large number of patients, examining 26 studies with a mean follow-up of 16 months, the overall success rate, complications rate, and incontinence rate were 76%, 14%, and 1.4%, respectively, with risk factors for recurrence being the existence of a horseshoe fistula, anal CD, and previous anal surgery.

The LIFT procedure may also be used [40] in anal CD; in a recent study with a long follow-up period, the healing rates were at 65%, and the majority of patients already had a seton placement before LIFT. LIFT gains a position in the treatment of complex transsphincteric AF in the recent guidelines of the ASCRS with a strong recommendation of evidence 1B.

Transanal Opening of the Intersphincteric Space (TROPIS)

It is a novel surgical sphincter-saving technique [41], the newest in the literature since 2017, with reported healing rates >90% without postoperative incontinence for complex AF. It has good long-term outcomes in follow-up. The procedure is suitable for high-grade complex AF [42] as well as supralevator ones. TROPIS is also recommended for horseshoe AF.

The procedure is of intelligent design, easily performed, and focused on treating surgically the intersphincteric fistulous tract. It involves laying open the fistula tract and secondary healing. The method competes with LIFT [43]. Both techniques, LIFT and TROPIS, present optimal surgical results with different surgical approaches; LIFT is focused on the closure of the IO of the fistulous tract, and TROPIS opens the intersphincteric space.

In a systematic review article on the efficacy and safety of sphincter-saving techniques [44] in complex AF, TROPIS may have the highest cure rates. The TROPIS represents a variation of the old Parks’ technique which requires to be tested by more surgeons and longer follow up in order to find its position in current practice and the guidelines.

Stem Cells Therapy of AF

This is an expensive treatment that is only suitable for highly selected patients with refractory anorectal fistulizing CD without any response to other conventional therapies. There are few reports in the literature with a limited number of patients.

In a multi-centric study [45] from six Spanish hospitals with 24 patients, there was an improvement in AF drain, with complete healing at 30%; the method could be repeated in a few weeks if active AF remains. In another multi-centric European study [46], there was significant local control and remission of the disease in 50% of patients; complications reported were proctalgia and anal abscess.

New minimally invasive sphincter-saving techniques for complex AF treatment

OTSC Proctology Device

It is a new endoscopic sphincter-saving technique [47] for the closure of the IO of the AF with a nitinol closure clip. The technique may be used in combination with other techniques such as VAAFT and fistula laser closure (FiLac) with a synergic effect.

Small studies exist, with a small number of patients and without long-term results. In a case study with 22 patients and long-term follow-up at three years [48], the complete healing rate of AF was 59%, the majority of clips were removed after 3-12 months (mean time of 5.8 months), and few clips remained in situ. No incontinence was reported, and one patient presented with burning after defecation and after the removal of the clip. The recurrence rate with active AF was 41%.

Other case studies with a small number of patients report higher rates of healing at 70% [49]. The procedure was performed in refractory AF with previous surgery, including patients with anal CD.

Another study reports healing rates [50] of 79% (short-terms outcomes) when the procedure was performed as first-line therapy, with lower healing rates at 45% and 20% in AF due to inflammatory bowel diseases and recto-vaginal fistulas, respectively. Indeed success rates in recurrent cases were 26%. The method cannot be evaluated at the moment, without any position in guidelines for AF therapy.

FiLaC Therapy

A fistula tract ablation technique with an approximate success rate of 60% or any more. Outcomes are better when the fistulous tract is of short length (3 cm) [51] with success rates at 58.3%, while in long fistulous tracts (>3 cm), the success rates decrease to 16.6%. The procedure does not treat secondary tracts or abscess cavities.

In another recent retrospective study [52] with AF of crypto-glandular etiology with 175 patients and long follow-up, healing rates at 66.8% is reported. In the majority of patients (81.8%) a seton was placed before FiLac for a mean period time of 14 weeks. Routine MRI or EUS before FiLac therapy was performed to exclude abscess cavities (contraindication for FiLac). The healing rates ranged from 51.5% up to 70.4% in patients with seton placement prior to FiLac therapy. Suprasphincteric AF was more difficult to treat, with a longer operative time. The method did not treat the IO; when it was enlarged due to seton, it was closed by some stitches in a few patients. The method as a noninvasive technique has also been used in anal CD [53], with a few cases reported in the literature.

In a meta-analysis [54] for the safety and efficacy of FiLac, with a two-year follow-up in transsphincteric and also recurrent AF in 454 patients, the healing rate, complication rate, and incontinence rate were 67.3%, 4%, and 1%, respectively.

In another meta-analysis [55] of the effectiveness of the FiLac, with 476 patients, 60% of whom had previous anal surgeries, the success and complication rates were 63% and 8%, respectively.

Despite the new technique of FiLac therapy for AF, new emerging data [56] exist regarding the variations of the technique, the selection of patients, outcomes, and complications. To date, the procedure may be performed in simple and complex AF, in anal CD, and in recurrent AF. The method may be repeated. Another similar new laser procedure is photo dynamic therapy (PDT) [57] with success rates of 80% with few cases reported in the literature at the moment.

The minimally invasive nature and the possibility of repeating the procedure have made it attractive to surgeons and patients and it has earned a position in the newest guidelines (2022) of the ASCRS with weak recommendation 2C, mainly due to lack of long-term outcomes. No recommendation in the oldest guidelines of the ACGBI and German S3 guidelines.

VAAFT

It is a new sphincter-saving technique without any significant postoperative complications or incontinence that has high healing rates. It is the only endoscopic procedure with visual access into the lumen of the fistulous tract. It provides the opportunity to concurrently treat the IO, secondary tracts, and abscess cavities with ablation of the fistulous tract.

The VAAFT procedure was proposed by Meinero et al. [58], who presented outcomes in 136 patients with complex AF in 2011, excluding CD and simple AF. The healing rate was 87.1% one year after surgery. Three years later, in a book chapter by the same author [59], based on 203 patients with complex and recurrent AF, the advantages of VAAFT were claimed to be: a large number of patients did not require preoperative assessment of fistula anatomy by MRI. In 59.6% of patients, secondary infections and abscesses were treated simultaneously and successfully during the procedure. Easy and rapid detection of the IO was achieved within five minutes in most patients (84.2%); in the remaining patients, it was detected by viewing the light of the fistuloscope inside the lumen of the anorectal canal. There was a short operative time of a median 30 minutes. The majority of patients had prior surgery, and the healing rates were 93.2% with a one-year follow-up. There were only minimal postoperative complications, which were treated conservatively. There was no incontinence. The majority were discharged on the same day of surgery. The lost time from work was up to three days.

The procedure rapidly gained popularity worldwide; it is also expanded [60] in the pediatric population and anal CD, which may be combined [61] with RAF. There is [62] a palliative effect in the main local anal symptoms, such as pain and discharge, in patients with anal CD and complex refractory AF undergoing VAAFT. At the moment, cases of anal Crohn’s disease reported in the literature are few, and if the equipment is available, the technique is expanded to other surgical entities [63], such as the pilonidal sinus.

In a case study [64] with 41 patients with complex cryptoglandular AF, the primary healing rate was 70.7%, the method was repeatable in recurrences, and the secondary healing rate was 83% using various techniques for the closure of the IO; closure with RAF had more recurrences than stapler closure, and OTSC had no recurrences but with a small number of patients.

In another case study [65] with 104 patients (those with secondary etiologies were excluded) and a three-year follow-up, the success rate was 84.4%. Recurrences were treated by the same procedure and were completely healed; the only risk factor for recurrence was the advanced age (>50 years).

In 2017, there was the first meta-analysis [66] on the efficacy of VAAFT in cryptoglandular complex AF, with success rates of 76%, minimal incontinence, a short hospital stay, and low complication rates.

In a recent systematic review and meta-analysis of the efficacy and safety of VAAFT [67], the recurrence rate was 14.2% with a follow-up of nine months, probably related to previous AF surgery and the method of closure of the IO of the fistulous tract; there were fewer recurrences after the use of staplers or sutures than in closure with RAF. The complication rate was 4.8%, the majority was of Clavien-Dindo severity score I or II, and no incontinence was reported in any study of this systematic review.

The latest meta-analysis [68] reports success, recurrence, and postoperative complications rates of 83%, 16%, and 11%, respectively, and recurrent cases vary according to the method of closure of the IO; the closure of the internal opening of the fistulous tract may be performed in several ways, and the RAF closure may be more susceptible to recurrences than other methods of closure. This observation may be biased as the advancement flap is used in insecure local conditions for the closure of the internal opening, while other, more simple techniques are used in safe local conditions. To date, there are no guidelines for the preferable option for the closure of the IO of the AF; local anal conditions around the internal opening, such as extensive fibrosis due to previous surgery or an inactive IO, should be well estimated for a secure closure; linear or semicircular staplers, sutures, OTSC or LIFT combined with VAAFT is a solution. If the closure is insecure, then an advancement flap is the last solution.

## Conclusions

During treatment for AF, there are a wide range of surgical procedures and a wide range of different anal conditions. Twelve surgical procedures are currently in use for the AF treatment, with an emphasis on new sphincter-saving procedures with high healing rates >60-80% and without any significant position in guidelines as they are in their infancy. Indeed, high diversity in local anal conditions exists regarding the grade of complexity and the existence or not of risk factors for recurrence such as secondary tracts, abscess cavities, horseshoe tracts, anterior AF in women, and anal trauma (previous anal surgery); these factors should be detected and treated during surgery. The etiology is another factor that increases the diversity of patients. Finally, surgery has multiple goals. First, the surgeon should eliminate during surgery any detected risk factor for recurrence. Second, in the era of sphincter-saving techniques, one should respect the anatomy and integrity of the anal sphincter system to avoid postoperative continence problems, and finally, one should choose a suitable procedure to treat the fistulous tract and assure a safe closure of the internal opening. The next point is the most crucial in the decision-making process for further surgery; after studying the complexity, a safe fistulotomy or sphincter-saving procedure should be used, where literature and guidelines leave several options for treatment. An experienced anal surgeon should assure a safe closure of the internal opening by choosing the most suitable procedure; many ways exist, such as laser therapy, VAAFT, LIFT, OTSC, RAF, and semicircular staplers. This concept of a safe closure of the internal opening has dominated for decades as the principal rule in the treatment of anal fistulas. On the other hand, new operations such as TROPIS recommend a lay-open technique with excellent surgical outcomes. This operation is the newest in literature and tries to find its position in the international surgical community and guidelines. More studies and literature data are needed to determine the final position of the newest techniques, such as laser therapy, VAAFT, OTSC, and TROPIS. This review study emphasizes new surgical techniques as they present optimal surgical results in the literature without any significant position in guidelines and probably will be the preferred procedure next year.
